# Intractable Ascites Management: The Role of Side-to-Side Portacaval Shunt

**DOI:** 10.1155/1999/47180

**Published:** 1999-04

**Authors:** J. Rodés

**Affiliations:** Liver Unit Hospital Clinic University of Barcelona Spain


[4] Yeo, C. J., Cameron, J. L., Lilemoe, K. D. *et al.* (1995). Pancreaticoduodenectomy for cancer of the head of the pancreas. 201 patients. *Ann. Surg.*, **221**, 721–733.
[5] Yeo, C. J., Cameron, J. L., Sohn, T. A. *et al.* (1997). Six hundred fifty consecutive pancreaticoduodenectomies in the 1990s: Pathology, complications, and outcomes. *Ann Surg.*, **226**, 248–260.

O. J. Garden, MD, FRCS (Glas & Ed)
Professor of Hepatobiliary Surgery
Director of Organ Transplantation
University Department of Surgery
Royal Infirmary, Lauriston Place
Edinburgh EH3 9YW United Kingdom

### Intractable Ascites Management: The Role of Side-to-Side Portacaval Shunt

### ABSTRACT

*Orloff, M. J., Orloff, M. S., Orloff, S. L. and Girard B. (1997) Experimental, clinical and metabolic results of side-to-side portacaval shunt for intractable cirrhotic ascites. The American College of Surgeons;* ***184****, 557–570.*

***Background:*** **Intractable ascites, refractory to medical therapy, occurs in approximately 10 percent of patients with ascites from cirrhosis and is almost always fatal. Sinusoidal hypertension resulting from hepatic venous outflow obstruction plays a primary role in the pathogenesis of cirrhotic ascites and provides the rationale for decompression of the liver by side-to-side portacaval shunt in treatment of intractable ascites. This report presents the experimental basis for the use of side-to-side shunt and long-term results of a prospective study in 34 selected patients with intractable cirrhotic ascites.**
***Study Design:*** **In the experimental studies, hepatic venous outflow obstruction and massive ascites were produced in dogs by ligation of the hepatic veins, and the effect of portacaval shunts on ascites, thoracic duct lymph flow, and aldosterone secretion were measured. In the clinical study, 34 carefully selected patients with cirrhosis (91 percent alcoholic) and truly intractable ascites (failure of medical therapy for 5 to 24 months) underwent side-to-side portacaval shunt. The effects on ascites, survival, metabolic abnormalities, and quality of life were studied prospectively during follow-up that was longer than 5 years in all but two patients. Quantitative Child's risk classes in percent of patients were A in 0, B in 68 and C in 32.**
***Results:*** **In the experimental studies, side-to-side portacaval shunt permanently relieved severe ascites, reduced the 13-fold increase in thoracic duct lymph flow rate to almost normal, and abolished the aldosterone hypersecretory response to minimal hepatic venous outflow obstruction. End-to-side portacaval shunt was much less effective. In the clinical study, side-to-side portacaval shunt reduced mean portal vein-inferior vena cava pressure gradient from 282 mm saline to 4 mm and permanently relieved all patients of ascites without subsequent requirement of diuretic therapy. Two patients who died of hepatoma, and one who died of heart failure developed terminal ascites. Thirty-day mortality rate was 6 percent and long-term survival rates at 5, 10 and 15 years were 75 percent, 74 percent and 73 percent. In metabolic studies, side-to-side shunt produced marked diuresis and natriuresis and abolished hypersecretion of aldosterone. Quality of life was generally improved as a result of a low incidence of recurrent portal-systemic encephalopathy (6 percent), abstinence from alcohol in 91 percent, improvement in liver function in 81 percent, and improvement in Child's risk class. The portacaval anastomosis remained permanently patent in every patient.**
***Conclusions:*** **Side-to-side portacaval shunt is very effective treatment of intractable ascites from cirrhosis. Our results are attributable to careful selection of patients, an organized system of care, and a program of rigorous, lifelong follow-up that emphasizes abstinence from alcohol and dietary protein restriction. (J. Am. Coll. Surg., 1997;** ***184*****, 557-570).**

*Keywords:* Portacaval shunt, side-to-side shunt, ascites, refractory ascites

### PAPER DISCUSSION

The development of refractory ascites in cirrhotic patients is relatively low. It is accepted that approximately 10 percent of the patients with ascites either do not respond to diuretic therapy or develop diuretic-induced complications that prevent the use of high doses of these drugs [1]. This condition is known as refractory ascites. Up to now the definition and diagnostic criteria of refractory ascites has not been well established. However, recently, in the biennial Meeting of the International Ascites Club held in Chicago in November 1994 [2], a group of experts defined this clinical condition. In this consensus conference it was considered that refractory ascites may be a consequence of diuretic-resistant or diuretic-intractable ascites. Diuretic resistant ascites was defined as ascites that cannot be mobilized or the early recurrence of which cannot be prevented due to a lack of response to sodium restriction (50 mEq/day sodium diet) and diuretic treatment (mean weight loss less than 200 g/day during the last four days of intensive diuretic therapy - spironolactone 400 mg/day and furosemide 160 mg/day- and urinary sodium excretion less than 50 mEq/day). Diuretic intractable ascites was defined as ascites that cannot be mobilized or the early recurrence of which cannot be prevented due to the development of diuretic-induced complications (hepatic encephalopathy, diuretic-induced renal failure, hyponatremia, and/or hypo or hyperkalemia), that preclude the use of an effective diuretic dosage. Moreover, most cirrhotics with refractory ascites have moderate renal failure or even steady hepatorenal syndrome (HRS). Although, it is possible that refractory ascites may be present in patients without renal failure, this situation is very uncommon.

The treatment of refractory ascites is still under discussion [2]. It is obvious that to establish therapy in any clinical condition a rationale is required. In cirrhotic patients with ascites this should be based on the pathophysiology of ascites formation and HRS. At present, it is considered that most of the available data regarding the development of renal functional abnormalities and ascites and edema formation in cirrhosis are related to the presence of an intense arteriolar vasodilattation (Arterial vasodilatation hypothesis) [3]. This hypothesis is based on the fact that arterial hypotension is frequent a finding in cirrhotics with ascites, despite an increased plasma volume and cardiac index, and a stimulated renin-angiotensin-aldosterone system (RAAS), sympathetic nervous system (SNS) and anti-diuretic hormone (ADH) that are all powerful vasoconstrictors. In addition, the blockade of the vascular effect of angiotensin II and ADH in cirrhosis with ascites is associated with a marked fall in peripheral vascular resistance and arterial pressure, an effect not observed in normal subjects. Therefore, by considering all these data, it could be suggested that the initial event in ascites formation is sinusoidal portal hypertension leading to marked arterial vasodilatation located mainly in the splanchnic circulation. Potential substances involved in this vasodilatation include nitric oxide and vasodilator peptides. Arterial vasodilatation leads to an abnormal distribution of blood volume with reduction of effective arterial blood volume and subsequent renal sodium and water retention due to the activation of vasoconstrictor systems. In the preascitic stage the retained fluid volume would suppress sodium and water retention and reestablish fluid balance at an upper level of blood volume. However, as the disease progresses, more vasodilatation occurs in the splanchnic circulation and the effective arterial blood volume can no longer be maintained by the increased blood volume, probably because fluid starts leaking from the splanchnic circulation to the peritoneal cavity as ascites. Then, a persistent activation of vasconstrictors and anti-natriuretic systems occurs in an attempt to maintain a normal effective blood volume and arterial pressure. The continuous leakage of intravenous fluid to the peritoneal cavity may explain the paradox of unrelenting activation of RAAS, SNS and ADH in the setting of an increased extracellular fluid volume. In very advanced stages of the disease, the disturbances in the splanchnic circulation are so marked that the systemic hemodynamics can be maintained only at the expense of vasoconstriction in most vascular beds, including the renal circulation, and therefore HRS develops.

At present, there is no real effective therapy for patients with refractory ascites. During the last two decades several therapeutic measures have been applied, and the rationale of the therapy is to reduce portal pressure, to increase the effective arterial blood volume or to improve renal perfusion.

Portacaval anastomosis, either side-to-side or end-to-side, was used in the past in the management of patients with refractory ascites. However, although this technique is very useful in controlling ascites, it has an associated high incidence of hepatic encephalopathy [4–6]. More recently Franco *et al.* [7] reported the results of portacaval shunt (most with side-to-side type) in 57 cirrhotics (90 percent alcoholics) with refractory ascites. Forty-six patients were in Child's class B an 11 in class C. This procedure was very effective in controlling ascites in all patients but one. Three patients died during surgery. Hepatic encephalopathy was detected in 50 percent of the patients after operation. The survival rate at 1 and 3 years of follow-up were 72 percent and 36 percent, respectively. Orloff *et al.* [8] have recently reported the results obtained with side-to-side portacaval shunt in patients with refractory ascites. This report included 34 cirrhotic patients (91 percent alcoholics) that were submited to portacaval shunts because of the presence of refractory ascites (68 percent of the patients were in Child's B class and 32 percent in class C). The operative mortality was very low. Only 6 percent of the patients died during the 30 postoperative days. The survival rate at 5, 10 and 15 years were 75 percent, 74 percent and 73 percent, respectively. Hepatic encephalopathy was detected in only 6 percent of the patients.

The results of these two studies are, in part, contradictory. As indicated by Orloff and his associates the best results obtained in their series may be due to a rigorous lifelong follow-up, that emphazises abstinence from alcohol and dietary protein restriction. However, in the study of Franco *et al.* continued drinking was not related to a higher incidence of hepatic encephalopathy. Therefore, this difference in the incidence of hepatic encephalopathy is not easily understandable. The only possibility is that these two groups of patients had different degrees of hepatic impairment. Unfortunately, in both studies liver function was not reported in detail. The value of bilirubin, prothrombin time and albumin, not indicated in these studies, is very important to compare these two series of patients. In addition, in neither renal function was measured by BUN or plasma creatinine. This again is very important to know since, as indicated, many patients with refractory ascites also have HRS. Moreover, the criteria used to indicate portacaval anastomosis was very imprecise, since no diuretic requirement and diet of sodium restriction to define refractory ascites was clearly established. Finally, in these two studies the time required to include 57 patients in Franco's report and 34 patients in the Orloff study was 14 and 33 years, respectively. This data clearly indicate that the patients with refractory ascites who can benefit by portasystemic shunt are very uncommon. Nowadays, it is difficult to ascertain whether this technique is the best procedure for these patients.

There are other therapeutic measures that have been used in the treatment of patients with refractory ascites. Peritoneovenous shunting (PVS) in ascitic patients induces a sustained expansion of the circulatory blood volume, suppresses RAAS, SNS and ADH, improves renal function and increases responsiveness to diuretics [1]. Unfortunately, PVS is associated with a high rate of complications such as shunt occlusion, that occurs in more than 40 percent of the patients at one year of follow-up, vena cava thrombosis and peritoneal fibrosis. At present, the indication of PVS has been markedly reduced, because of the re-introduction of therapeutic paracentesis [1]. In fact, this technique, associated with intravenous administration of albumin, has been widely used in the management of ascites and in the treatment of patients with refractory ascites. A controlled clinical trial was performed to compare therapeutic paracentesis vs PVS [1]. The results obtained indicate that, although PVS was better than paracentesis in the long-term control of ascites, it does not reduce the total time in hospital during follow-up or increase survival. In addition, most patients needed to be reoperated upon because of shunt obstruction. At present, in many centers therapeutic paracentesis associated with intravenous albumin infusion is used in the management of patients with refractory ascites and those considered as candidates for liver transplantation.

A number of studies have recently been published investigating the efficacy of transjugular intrahepatic portasystemic shunt (TIPS) in the management of patients with refractory ascites [9]. TIPS functionally acts as a side-to-side portacaval shunt. It produces a reduction in portal pressure and is associated with favorable effects on renal function with elimination or decrease of ascites. The main advantage of TIPS over surgical portasystemic shunts is that this technique is less aggressive and probably the operative mortality is lower. However, obstruction is common and leads to accumulation of ascites. Other complications observed are the development of hepatic encephalopathy and impairment of liver function due to the shunting of blood from the liver to the systemic circulation. A recent comparative study in a small series of patients with refractory ascites showed increase mortality in patients treated with TIPS, especially in patients with poor liver function, compared to patients treated with paracentesis plus albumin [10]. Therefore, larger controlled clinical trials are required to define the role of TIPS in the management of cirrhotic patients with refractory ascites.

A variety of drugs has been used in patients with refractory ascites and HRS with only minor or no effects. Recent studies suggest that the administration of systemic vasoconstrictors, particularly ornipressin, combined with plasma expansion with albumin for a prolonged period of time (up to 15 days) may markedly improve renal function in patients with HRS [11]. However, it should be taken into account that this therapy may be associated with ischemic complications. Moreover, it is not known whether the improvement of renal function has a favorable effect on survival. Therefore, the usefulness of this therapy should be evaluated in prospective controlled investigations.

Without doubt the treatment of choice in patients with refractory ascites in selected candidates is liver transplantation [12]. However, a significant proportion of patients die before transplantation can be performed because of their extremely short survival rate. Therefore, liver transplantation should be indicated, whenever possible, before the development of refractory ascites. Patients most likely to develop refractory ascites are those with marked sodium and water retention, dilutional hyponatremia and marked activation of vasoconstrictor systems.

In conclusion, although the Orloff report is very interesting and is in favor of portasystemic shunts in the management of refractory ascites, there are several aspects that should be taken in account before considering what the best procedure for these patients is. The most important concerns of this report are the following: 1) There is no a clear definition of refractory ascites, 2) There is no data on renal and liver function before the indication of the operation, 3) There is no data on liver histology before surgery to permit assessment whether these patients had underlying hepatic cirrhosis, associated or not, with alcoholic hepatitis and finally, 4) The number of patients in whom portacaval shunts was indicated was so small (1 patient per year) that this therapy should be considered as anecdotal and only useful in very selective patients.

### References

[1] Ginés, P., Arroyo, V., Vargas, V., Planas, R, Casafont, F., Panés, J., Hoyos, M., Viladomiu, L., Rimola, A., Morillas, R., Salmerón, J. M., Ginés, A., Esteban, R. and Rodés, J. (1991). Paracentesis with intravenous infusion of albumin as compared with peritoneovenous shunting in cirrhosis with refractory ascites. *New England Journal of Medicine*, **13**, 341–343.

[2] Arroyo, V., Ginés, P., Gerbes, A. L., Dudley, F. J., Gentilini, P., Laffi, G., Reynolds, T. F., Ring- Larsen, H. and Schölmerich, J. (1996). Definition and diagnostic criteria of refractory ascites and hepatorenal syndrome in cirrhosis. *Hepatology*, **23**, 164–176.

[3] Schrier, R. W., Arroyo, V., Bernardi, M., Epstein, M., Henriksen, J. H. and Rodés, J. (1988). Peripheral arteriolar vasodilatation hypothesis a proposal for the initiation of renal sodium and water retention in cirrhosis. *Hepatology*, **8**, 1151–1157.

[4] Barker, H. G. and Reemtsma, K. (1960). The portacaval shunt operation in patients with cirrhosis and ascites. *Surgery*, **48**, 142–154.

[5] Welch, H. F., Welch, C. S. and Carter, J. H. (1964). Prognosis after surgical treatment of ascites: Results of side-to-side shunt in 40 patients: *Surgery*, **56**, 75–82.

[6] Burchell, A. R., Rousselot, L. M. and Panke, W. F. (1968). A seven-year experience with side-to-side portacaval shunt for cirrhotic ascites. *Annals of Surgery*, **168**, 655–670.

[7] Franco, D., Vons, C., Traynor, O. and Smadja, C. (1988). Should portasystemic shunt be reconsidered in the treatment of intractable ascites in cirrhosis? *Archives of Surgery*, **123**, 987–991.

[8] Orloff, M. J., Orloff, M. S., Orloff, S. L. and Girard, B. (1997). Experimental, clinical and metabolic result of side-to-side portacaval shunt for intractable cirrhotic ascites. *Journal of the American College of Surgeons*, **184**, 557–569.

[9] Ochs, A., Rössle, M., Haag, K., Hauenstein, K. H., Deibert, P., Siegerstetter, V., Huonker, M., Langer, M. and Blum, H. (1995). The transjugular intrahepatic portasystemic stent-shunt procedure for refractory ascites. *New England Journal of Medecine*, **332**, 1192–1197.

[10] Lebree, D., Giuily, N., Hadengue, A., Vuilgrain, V., Moreau, R., Poynard, T., Gadano, A., Lassen, C., Benhamou, J. P. and Erlinger, S. (1996). Tansjugular intrahepatic portasystemic shunts comparison with paracentesis in patients with cirrhosis and refractory ascites: a randomized trial. *Journal of Hepatology*, **25**, 135–144.

[11] Guevara, M., Ginés, P., Fernández-Esparrach, G., Sort, P., Salmerón, J. M., Jiménez, W., Arroyo, V. and Rodés, J. (1998). Reversibility of hepatorenal syndrome by prolonged administration of ornipressin and plasma volume expansion. *Hepatology*, **27**, 35–41.

[12] Gonwa, T. A., Morris, C. A., Goldstein, R. M., Husberg, B. S. and Klintmalm, G. B. (1991). Long-term survival and renal function following liver transplantation in patients with and without hepatorenal syndrome. Experience in 3000 patients. *Transplantation*, **51**, 428–430.

Prof. J. Rodés, MD
Liver Unit
Hospital Clinic
University of Barcelona
Spain

### Does Sphincteroplasty Predispose to Bile Duct Cancer?

### ABSTRACT

*Hakamada, K., Sasaki, M., Endoh, M., Itoh, T., Morita, T. and Konn, M. (1998) Late development of bile duct cancer after sphincteroplasty: A ten- to twenty-two-year follow-up study. Surgery; 121, 488–492.*

*Background:* **Transduodenal sphincteroplasty is designed to destroy the sphincteric muscle fibers, producing a terminal choledochoduodenostomy. In the absence of Oddi's sphincter, intestinal contents with both activated pancreatic juice and bacterial flora are refluxed into the bile duct and remain there for a prolonged time. The long-term effect of producing the reflux has not been evaluated to date.**

*Methods:* **One hundred nineteen consecutive patients undergoing transduodenal sphincteroplasty between February 1973 and July 1984 were included in this study. Postoperative clinical courses of 108 patients could be evaluated by means of a retrospective review of the hospital records. Median follow-up was 18 years.**

*Results:* **Eight cases (7.4%) of primary bile duct cancer were found among the 108 cases at intervals of 1 to 20 years after sphincteroplasty. Two patients has concurrent hepatolithiasis. The patency of sphincteroplasty was confirmed in all cases, and the bile was infected in seven cases. Pathologic specimens obtained demonstrated cholangiocarcinomas and various degrees of atypical hyperplastic lesions under the background of chronic cholangitis.**

*Conclusions:* **Chronic cholangitis can be an important causative factor in late development of bile duct cancer after sphincteroplasty. Any patients treated with choledochoduodenostomy should be closely monitored for late cholangiocarcinoma. (*Surgery*, 1997; *121*: 488–492).**

